# Aerodynamic/Hydrodynamic Investigation of Water Cross-Over for a Bionic Unmanned Aquatic–Aerial Amphibious Vehicle

**DOI:** 10.3390/biomimetics9030181

**Published:** 2024-03-17

**Authors:** Wenbiao Gan, Zhenjie Zuo, Junjie Zhuang, Dawei Bie, Jinwu Xiang

**Affiliations:** 1 Institute of Unmanned System Research, Beihang University, Beijing 100191, China; ganhope@buaa.edu.cn; 2 School of Aeronautic Science and Engineering, Beihang University, Beijing 100191, China; bjutzuozj@sina.com (Z.Z.); 19377094@buaa.edu.cn (J.Z.); 3 Tianmushan Laboratory, Yuhang District, Hangzhou 311115, China; biedawei1@163.com

**Keywords:** water cross-over, aerodynamic/hydrodynamic, aquatic-aerial amphibious vehicle, bionic design, numerical simulation, experiment investigation

## Abstract

An aerodynamic/hydrodynamic investigation of water cross-over is performed for a bionic unmanned aquatic–aerial amphibious vehicle (bionic UAAV). According to flying fish features and UAAV flight requirements of water cross-over, the bionic conceptual design of crossing over water is described and planned in multiple stages and modes of motion. A solution procedure for the numerical simulation method, based on a modified SST turbulence model and the VOF model, is expressed, and a verification study is presented using a typical case. Longitudinal–lateral numerical simulation analysis investigates the cruise performance underwater and in the air. The numerical simulation and principal experiment verification are conducted for crossing over water and water surface acceleration. The results indicate that the bionic UAAV has an excellent aerodynamic/hydrodynamic performance and variant configuration to adapt to water cross-over. The bionic UAAV has good water and air navigation stability, and the cruise flying lift–drag ratio is greater than 15 at a low Reynolds number. Its pitching moment has the phenomenon of a “water mound” forming and breaking at the water cross-over process. The present method and the bionic variant configuration provide a feasible water cross-over design and analysis strategy for bionic UAAVs.

## 1. Introduction

In the wake of developments in aircraft design and bionic mechanical science, bionic unmanned aquatic–aerial amphibious vehicles (bionic UAAVs) with variant configurations have gradually become one of the research hotspots [1,2,3,4]. Based on vehicle morphology and motion control ideas of natural organisms, the bionic UAAV can imitate their biological characteristics and configurations, which adapt to excellent amphibious survival characteristics in water and air. In order to maintain efficient amphibious performance, the bionic UAAV should have efficient characteristics of water cross-over. Therefore, the aerodynamic/hydrodynamic investigation of water cross-over can significantly improve efficiency and performance and provide practical significance for designing and analyzing bionic UAAVs [5].

According to the different characteristics of biomimetic organisms across natural water, the bionic UAAV can be divided into seabird-like and fish-like. The seabird-like UAAV has high flight performance, but underwater endurance and water cross-over stability are limited. The fish-like UAAV has the characteristic of good impact performance during water and underwater cross-over processes, but its flight performance is relatively weak. Therefore, starting with fish-like UAAVs, combined with sufficient characteristics of variant aircraft, the configuration concept design and aerodynamic/hydrodynamics are necessary to investigate the water cross-over of the bionic UAAVs. It is highly feasible and promising for the research field of bionic UAAVs.

In bionic design, Pena designed a gannet-inspired amphibious drone [6]. Yun was inspired by the bionic kingfisher [7]. Lu employed a model that combined the kingfisher and diving beetle [8]. Geder designed a flying-swimmer UAV [9,10]. Yang created a squid-like UAAV [11]. This research focuses on mechanical practice, and their inspiration comes from different animals. Regarding numerical simulation, Lu utilized a dynamic mesh to analyze the variations in hydrodynamic forces as the water exit angle and velocity changed [9]. Wick examined the impact characteristics of various inlet lip configurations [12]. Pena performed a computational analysis using the panel method [6]. Yang combined the VOF model with Navier–Stokes equations to simulate the water-entry and underwater process [13,14]. Siddall conducted wind and water tunnel experiments in the experimental study to investigate the dynamic water–air performance of bionic UAAVs [3]. Backer experimented with water impact on axisymmetric objects [15]. Lock verified the effect of marine locomotion constraints on a bio-inspired aerial–aquatic wing [16]. These studies have primarily focused on single-scene configurations, resulting in limited bionic UAAV performance.

Since the idea of a flying submarine in 1934, there have been four significant UAAV concepts: the LPL prototype, the RFS-1 prototype, the Convair prototype, and DARPA’s submersible aircraft [17,18]. However, insufficient attention has been given to comprehensively examining water cross-over’s distinct stages and flight patterns for bionic UAAVs. Therefore, modeling these stages and providing a numerical simulation analysis of the whole process are imperative for water cross-over. Furthermore, practical experiments should be conducted to validate the morphology and motion control of the applicable models.

This paper examines the aerodynamic and hydrodynamic features of a bionic UAAV in motion. The bionic design was created in stages to achieve successful flight over water, including underwater navigation, acceleration on water, and balanced aerial flight inspired by flying fish. To verify the design, a numerical simulation method utilizing a modified Shear Stress Transport (SST) turbulence model and the Volume of Fluid (VOF) model was employed and tested through a typical case. The longitudinal–lateral numerical simulation analysis explored the bionic UAAV’s underwater and air cruise performance, showing excellent aerodynamic and hydrodynamic performance with appropriate water and air navigation stability. The results also revealed a “water mound” forming and breaking during the water cross-over process.

## 2. Bionic Conceptual Design of Water Cross-Over

A bionic conceptual design for a bionic UAAV is developed based on the characteristics of flying fish across water. The fins of these flying fish can be folded and extended to adapt to different environments. Numerous scholars have researched and designed these conceptual prototypes, combining them with flying fish’s efficient water entry and exit characteristics [19]. Some examples include the flying fish diving drone from Beihang University [20,21], the prototype booby with a hanging propeller from Beihang University [22,23], the tiny robot that imitates flying fish from Franklin W. Olin College of Engineering [24], the cormorant drone from Lockheed Martin [25], the drift and fly dual-mode flying fish drone from the University of Michigan [26,27], the mock squid jet drone from Imperial College London [28], the RoboBee prototype and insect-like flapping wing amphibious UAAV from Harvard [29,30], the MHAUV from Shanghai Jiao Tong University [31], and the Harrier and other vertically launched swept-wing reconnaissance drones and nail models [32].

1. During underwater diving, the pectoral fins and tail fins are folded against both sides of the body to reduce resistance and maintain stability;
2. While gliding in the air, the pectoral fins and tail fins are fully extended, resembling fixed-wing aircraft;
3. When crossing over the water, the pectoral fins extend only while the tail fins deflect, creating a configuration similar to the acceleration process on the water of the seaplane.

Considering the characteristics above, several stages can be identified for bionic conceptual design: an underwater diving process, a water cross-over process, an acceleration process on the water, and an air gliding process.

### 2.1. Bionic Fuselage Design and Modeling

**Figure 1:**
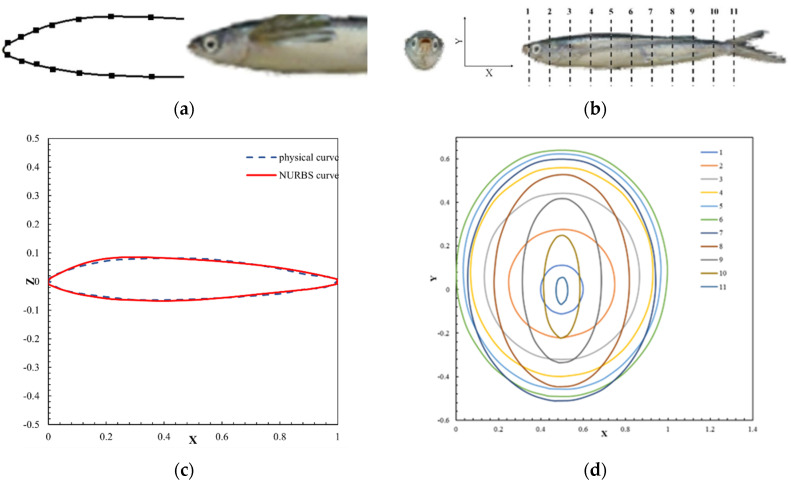
The bionic fuselage curves. (**a**) The longitudinal characteristics of flying fish; (**b**) the lateral characteristics of flying fish; (**c**) the longitudinal section curve of the fuselage generated by NURBS; (**d**) the lateral section curve of the fuselage caused by NURBS.

The longitudinal section of the UAUV, generated by Non-Uniform Rational B-Splines (NURBS), is described in Figure 1a,c. The practical sections are demonstrated in Figure 1b. According to the position of the control points, a series of dimensionless cross-sections is shown in Figure 1d, which has 11 sections and is dimensionless-sized by the maximum width of the body. However, the bionic UAAV model is determined by longitudinal and horizontal sections. These sections are then used for multi-section surface modeling to form a complete three-dimensional fuselage surface [33].

### 2.2. Configuration of Underwater Diving and Water Cross-Over Process

**Figure 2:**
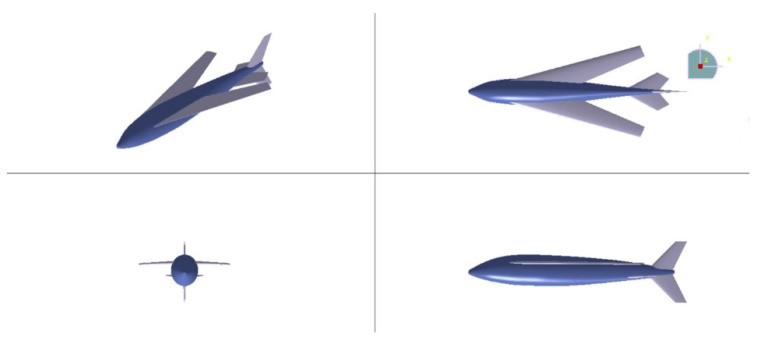
The schematic diagram of the underwater diving and water cross-over processes.

In underwater diving, the folding form of flying fish’s pectoral fins and tail fins is referenced and combined with the control requirements of bionic UAAVs. The wings and flat tail are folded backward, while some tail wings and main wings are positioned on both sides of the fuselage. This design not only facilitates navigation stability but also prevents spatial interference caused by the enfoldment of the wings. For the water cross-over process, flying fish swiftly cross-over the water in nature at a slight angle between 0° and 30°, subsequently accelerating on the surface by swinging tail wings. Only in dangerous situations do they rapidly cross over the water at a large angle ranging from 60 to 90 degrees. Before crossing the water, flying fish maintain their streamlined shape for underwater navigation. However, they quickly expand their pectoral and tail fins within one second after crossing the water. Therefore, the configuration of underwater diving and the water cross-over process of bionic UAAVs can emulate shapes similar to flying fish’s shallow-water diving process (referring to Figure 2) [34]. Table 1 and Table 2 provide the fuselage contour and configuration parameters of underwater diving and water cross-over processes.

### 2.3. Configuration of Acceleration Process on the Water

**Figure 3:**
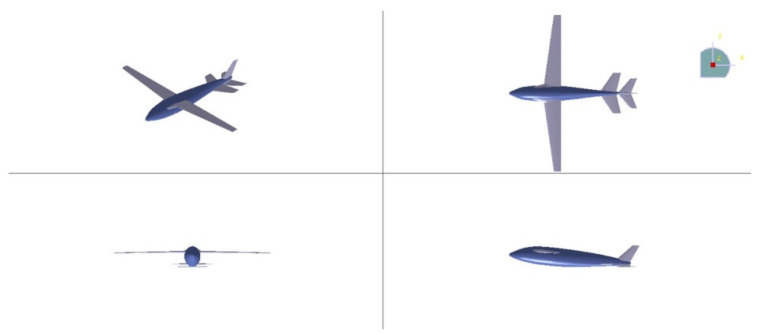
The schematic diagram of the acceleration process on the water.

In the acceleration process on the water, to ensure that the bionic UAAV achieves sufficient takeoff speed and stability, we incorporate the shape of flying fish and the acceleration characteristics of seaplanes. The wings and tail of bionic UAAV are fully extended, with an extended sagging tail on both sides to provide support similar to near-surface buoys. Figure 3 illustrates the schematic modeling of the acceleration process on the water. Table 3 provides the configuration parameters of the acceleration process on the water and air-balanced flight.

### 2.4. Configuration of Air-Balanced Flight

**Figure 4:**
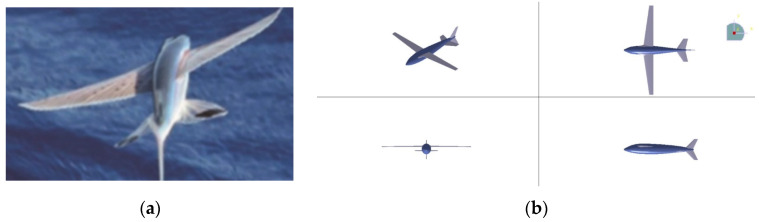
The schematic diagram of the air-balanced flight process. (**a**) Actual image of flying fish in flight; (**b**) modeling for air-balanced flight.

Inspired by the actual image of a flying fish in flight at a speed of 10 m/s, we optimized the lift and lift–drag ratio by spreading all wings and using a flat tail design resembling the tail of a flying fish during the air-balanced flight process. The comparison of organisms and the bionic UAAV is depicted in Figure 4. Configuration parameters of acceleration process on the water and air-balanced flight can be found in Table 4

## 3. The Numerical Methods

### 3.1. The Modified SST Model

The k-ω Shear Stress Transport (SST) model employs a mixture function and modified dynamic viscosity function by Menter (1994). It can adapt to the k-ω model near the wall and the *k*-ε model away from it. The transport equations of the model include production items and eddy viscosity based on the magnitude of vorticity Ω ($\Omega =\sqrt{2\Omega_{ij}\Omega_{ij}}$). They are summarized as follows [35,36,37]:

(1) $$\begin{array}{ll} \frac{\partial \left(\rho k\right)}{\partial t}+\frac{\partial (\rho u_{j}k)}{\partial x_{j}} & =P_{k}-\beta^{\prime }\rho k\omega \\ & +\frac{\partial }{\partial x_{j}}\left[\left(\mu +\frac{\mu_{T}}{\sigma_{k}}\right)\frac{\partial k}{\partial x_{j}}\right] \end{array}$$

(2) $$\begin{array}{ll} \frac{\partial \left(\rho \omega \right)}{\partial t}+\frac{\partial (\rho u_{j}\omega )}{\partial x_{j}} & =P_{\omega }-\beta \rho \omega^{2}\\ & +\frac{\partial }{\partial x_{j}}\left[\left(\mu +\frac{\mu_{T}}{\sigma_{k}}\right)\frac{\partial \omega }{\partial x_{j}}\right]\\ & +2(1-F_{1})\frac{\rho }{\sigma_{\omega_{2}}\omega }\frac{\partial k}{\partial x_{j}}\frac{\partial \omega }{\partial x_{j}} \end{array}$$

The Reynolds stress-based and separation-based ideas modify Menter’s *k*-*ω* SST turbulence model.

### 3.2. The VOF Model

The VOF model is an interface capture method (Hirt & Nichols, 1981; Muzaferija, 1998). It employs function C, satisfying the transport equation, whose magnitude is the ratio of fluid–grid volume. The value of C equals 0. No medium is contained in a grid cell. In contrast, C equals one if a grid cell is filled with a medium. When cells have interfaces, C is between 0 and 1. By solving the transport equation of function C at any time, the distribution of the VOF function in the computational domain can be obtained, and the interface can be captured.

In this paper, the simulation method, based on the VOF model and dynamic mesh, uses the finite-volume method of the structure grid to solve the three-dimensional unsteady RANS (3D-URANS). The implicit LU-SGS splitting time and Roe flux-difference splitting spatial method are used. The SIMPLE algorithm solves the pressure–velocity coupling problem (Van Doormaal & Raithby, 1984). The second-order discrete is adapted to the momentum convection term and the turbulence convection and diffusion. The no-slip condition is employed for solid boundaries. The far-field boundary adopts a free-stream boundary condition [38,39,40].

### 3.3. Validation

**Figure 5:**
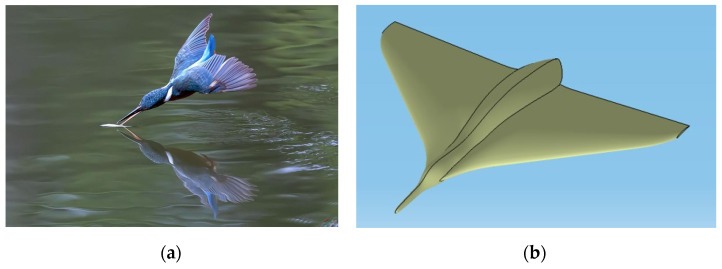
The bionic UAAV is based on a kingfisher and predacious diving beetle. (**a**) Actual kingfisher image; (**b**) modeling based on a kingfisher and predacious diving beetle.

For specific verification regarding turbulence simulation in numerical methods, please refer to Ref. [41]. To validate our adopted multiphase flow calculation method, we conducted numerical simulations based on water cross-over simulations of aircraft that imitate the kingfisher and Flying Snake in Ref. [42]. Our bionic UAAV model combines features from both species that utilize the kingfisher’s head and the body shape of a predacious diving beetle (referring to Figure 5).

**Figure 6:**
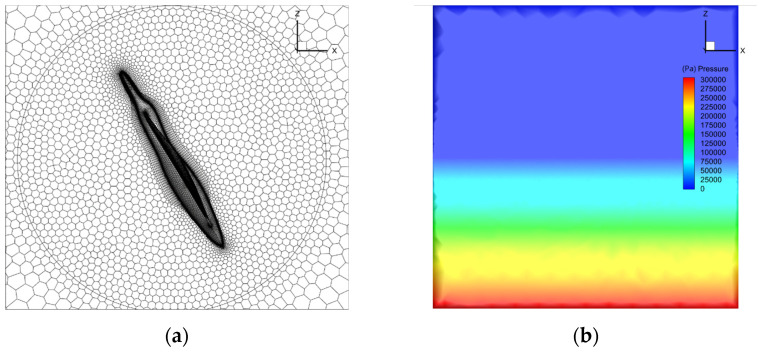
The schematic diagram of aircraft grid division and initial pressure distribution. (**a**) Grid division; (**b**) far-field initial pressure distribution.

To confirm the position of the water–air interface more precisely, we concentrated on the verification model of the flow field near the aircraft and the water–air interface. Meanwhile, poly-hexacore was adopted for division to minimize the consumption of computing resources, as shown in Figure 6a. The distance between the far field and the body was 50 times the body length. The boundary conditions were set as the body of a wall and the far field as the exit for pressure. The turbulence model adopted the SST k-w model, the multiphase flow model adopted the VOF model, and the grid movement mode adopted the overall method. The reflux volume fraction of the far field was set through the User-Defined Function (UDF) method to keep the water level constant, and the distant field pressure was corrected by the UDF method. The initialized far-field pressure distribution is shown in Figure 6b.

**Figure 7:**
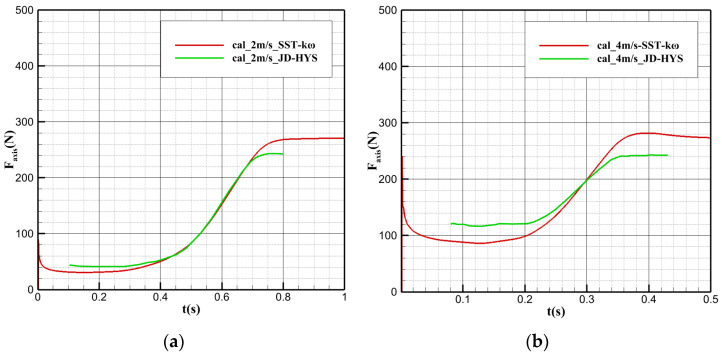
Axial force variation with time during the water cross-over process at different speeds. (**a**) v = 2 m/s; (**b**) v = 4 m/s.

Numerical simulation verification was conducted for the water cross-over angle at 60° with a constant speed of 2 m/s and 4 m/s. Figure 7 shows the comparison chart of the axial force with different water cross-over speeds against time, which is consistent with that in the literature. However, due to the influence of the difference in the initial moment, there is a minimal deviation between the two results. Before the bionic UAAV model contacts the water surface, the overall change in the axial force is relatively smooth. From contact with the water surface to complete deviation from the water, the axial force gradually and acutely changes. Then, it maintains an approximately constant increasing slope with a stable magnitude. After completely varying from the water surface, the axial force remains unchanged. Analyzing the axial force’s specific value after completely deviating from the water, the model is mainly subjected to the axial component of its gravity after varying from the water, which accounts for up to 99.54 percent, consistent with the results in Ref. [42].

**Figure 8:**
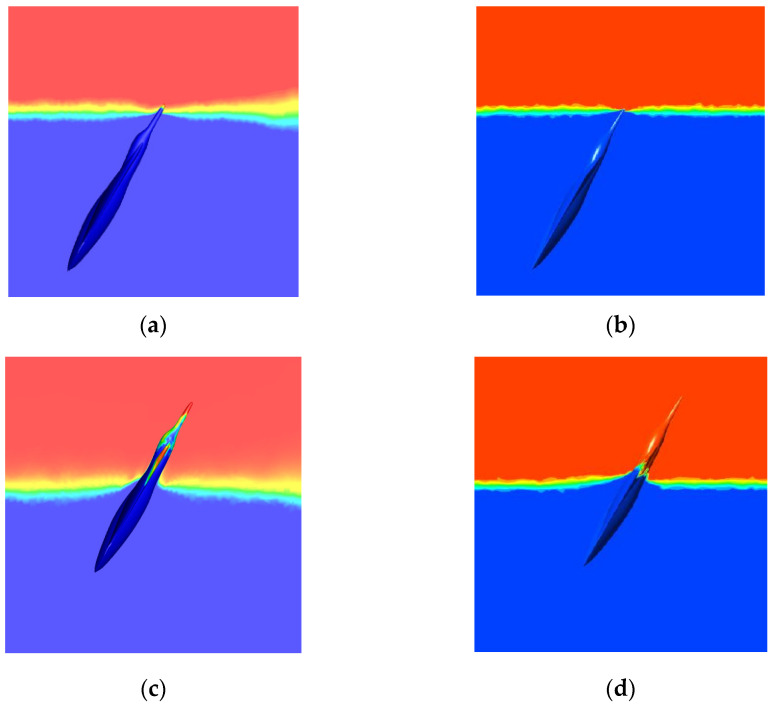

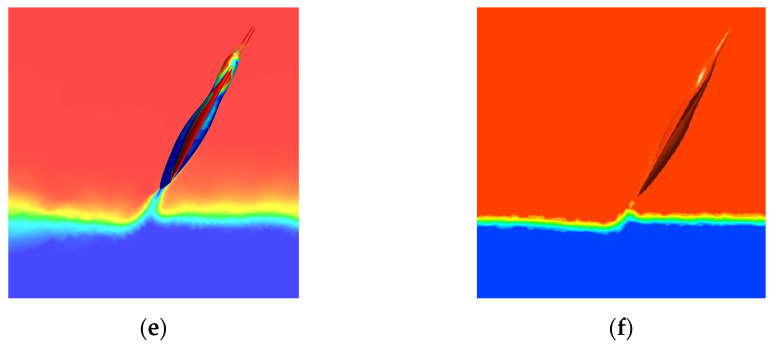
Comparison of phase distribution during the typical water cross-over process (α = 60°, v = 4 m/s). (**a**) Simulation results of critical water cross-over; (**b**) literature results of essential water cross-over; (**c**) simulation results of partial water cross-over; (**d**) literature results of partial water cross-over; (**e**) simulation results of complete water cross-over; (**f**) literature results of whole water cross-over.

Figure 8 compares phase distributions at different stages (water cross-over angle of 60°, speed of 4 m/s). According to varying degrees of the water cross-over process, this process can be divided into three phases: critical, partial, and complete. The crucial stage occurs when the model first makes contact with the water surface, while the partial water cross-over process occurs when the midpoint of the model’s body axis is close to or near the water surface. The complete water cross-over process separates the model and the water surface. Changes in the water level near the model during its crossing are consistent with Ref. [42]. The simulation results regarding liquid phase adhesion characteristics are more apparent than Ref. [42].

## 4. Performance of Underwater Navigation and Air-Balanced Flight

### 4.1. Longitudinal Hydrodynamic Performance of Underwater Navigation

**Figure 9:**
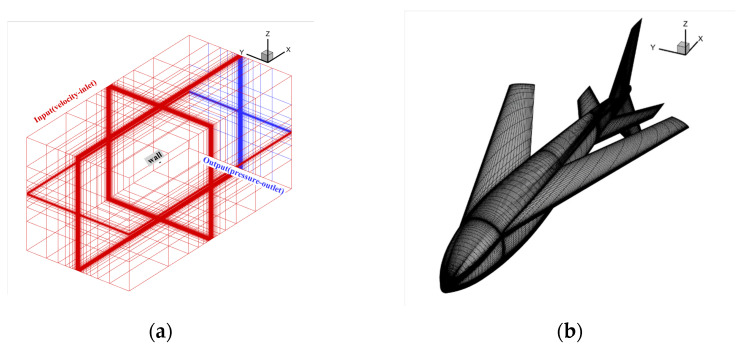
The schematic diagram of bionic UAAV grid division of underwater navigation. (**a**) Spatial grid; (**b**) airframe grid.

We calculated the whole machine’s longitudinal hydrodynamic characteristics for the underwater submersible process at two typical speeds of 0.5 m/s and 1 m/s. In this state, the wings are folded backward. Hence, the body length is chosen as the characteristic length. Therefore, the Reynolds numbers are Re1 ≈ 300,000 and Re2 ≈ 600,000, respectively, and the calculation angle of attack ranges from −6° to 10°. The calculation grid is shown in Figure 9 with the boundary condition of far-field pressure and a fixed non-slip wall.

**Figure 10:**
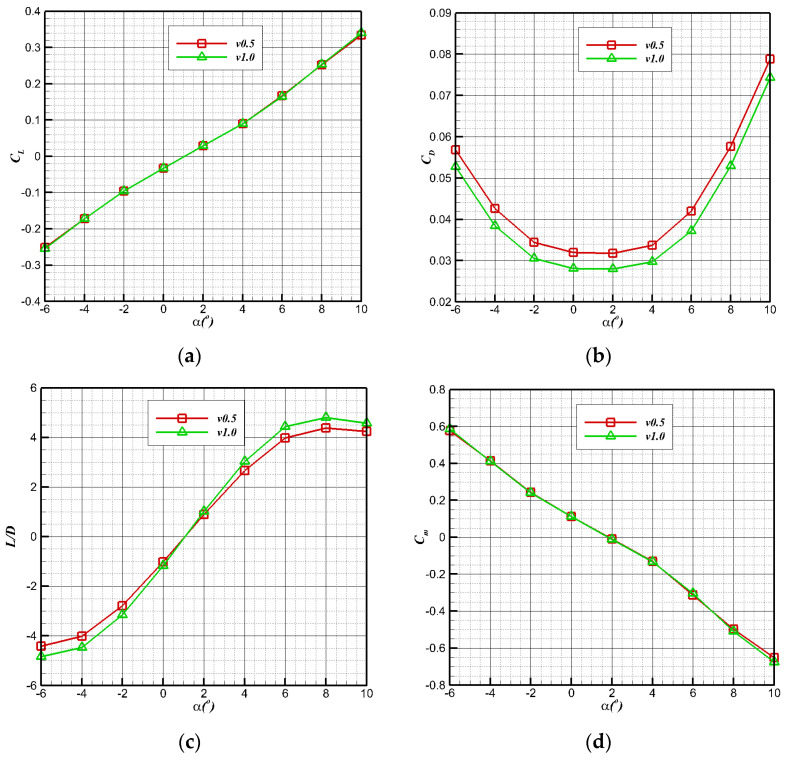
Longitudinal hydrodynamic performance of underwater navigation. (**a**) Lift coefficient; (**b**) resistance coefficient; (**c**) lift–drag ratio; (**d**) pitching moment coefficient.

Figure 10 shows the longitudinal aerodynamic characteristic curve of bionic UAAV underwater navigation. With the increase in the angle of attack at two speeds, the lift coefficient ($C_{L}=\frac{L}{\frac{1}{2}\rho {V_{\infty }}^{2}S}$) maintains linear growth, the whole resistance coefficient ($C_{D}=\frac{D}{\frac{1}{2}\rho {V_{\infty }}^{2}S}$) begins to rise sharply when the angle of attack is greater than 4°, and the lift–drag ratio (K=CLCD) rapidly decreases. At a speed of 0.5 m/s, the maximum lift–drag ratio is about 4.4, while at a speed of 1 m/s, the maximum lift–drag ratio is about 4.8. Finally, the maximum lift–drag ratio reaches the peak when the angle of attack is 8°. According to the change in pitching moment coefficient ($C_{m}=\frac{M}{qS_{w}c_{a}}$) curve, the vehicle has good pitching static stability at underwater diving speeds of 0.5 m/s and 1 m/s.

**Figure 11:**
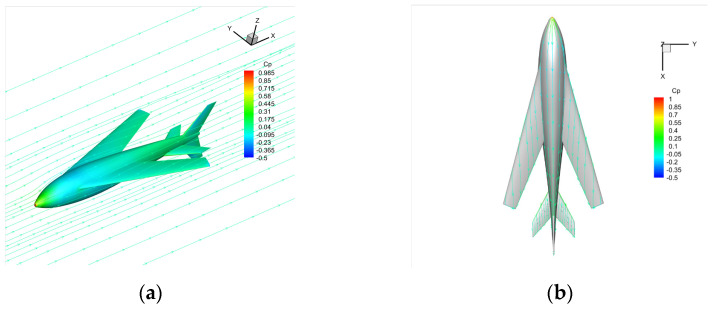
The schematic diagram flow field in the typical state (v = 0.5 m/s, α = 0°). (**a**) Space streamline; (**b**) surface limit streamline.

Figure 11 shows the characteristics of the flow field at a speed of 0.5 m/s and an attack angle of 0°. The space streamline offers the existence of a specific wing tip vortex. The surface limit streamline shows a large area of wing surface spreading flow, and attention should be paid to its impact on the pressure center and pitching stability.

### 4.2. Lateral Heading Performance of Underwater Navigation

**Figure 12:**
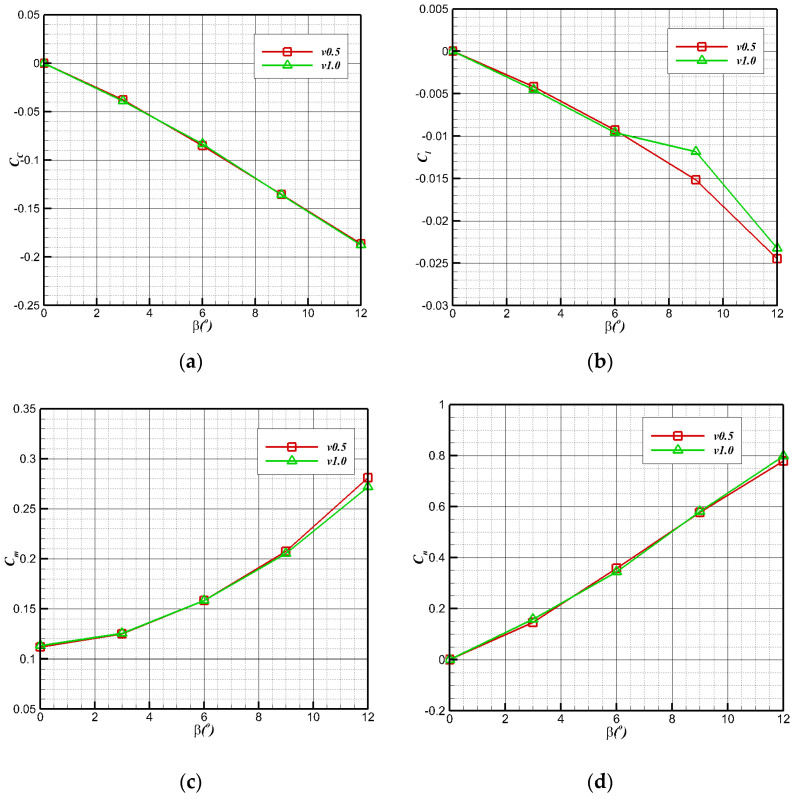
Lateral hydrodynamic performance of underwater navigation. (**a**) Lateral force coefficient; (**b**) roll moment coefficient; (**c**) pitch moment coefficient; (**d**) yaw moment coefficient.

To evaluate the lateral stability of underwater navigation of the bionic UAAV, calculation and analysis were conducted for α = 0° and β = 0–12° at a depth of 10 m (corresponds to the typical working environment of UAAV). The calculation results are shown in Figure 12. The yaw moment and lateral force coefficients show linear changes with the increase in sideslip angle. The bionic UAAV exhibits good lateral static stability.

**Figure 13:**
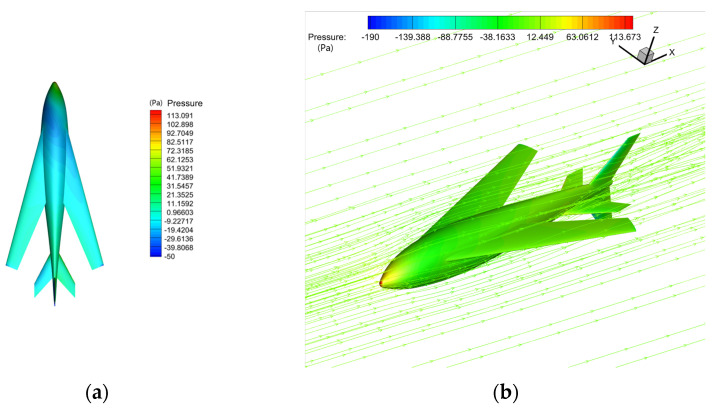
The schematic diagram flow field of a typical sideslip (v = 0.5 m/s, α = 0°, β = 6°). (**a**) Pressure distribution; (**b**) space streamline.

The flow field during a typical sideslip is shown in Figure 13 (v = 0.5 m/s, α = 0°, β = 6°). The asymmetric pressure distribution on the left and right sides of the wing, especially at the joint part of the wings and the body, will impact the roll moment directly. The spatial streamline diagram shows that the water flow can pass by the fuselage head relatively steadily under the influence of the sideslip angle. At the same time, there is a small range of flow separation in the vertical tail leeward area.

**Figure 14:**
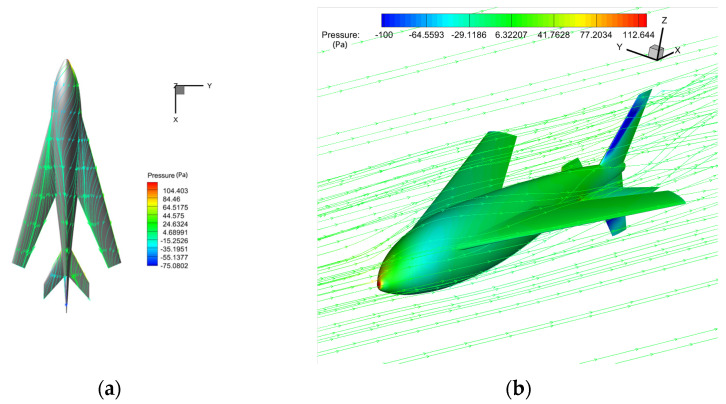
The schematic diagram flow field of large sideslip (v = 0.5 m/s, α = 0°, β = 12°). (**a**) Surface limit streamline; (**b**) space facilitate.

The typical flow field of a large sideslip is shown in Figure 14 (V = 0.5 m/s, α = 0°, β = 12°). The limit streamline distribution on the wing’s upper surface shows that the spanwise flow area at the leeward side is more significant than that at the leeward side. The aircraft induces an apparent wake vortex and necessary sideslip flow separation in the vertical tail leeward area. The asymmetric pressure distribution on the left and right sides of the wing and the wake vortex induction in the vertical tail leeward area will lead to significant changes in the lateral heading moment. At the same time, considerable sideslip flow separation will also aggravate this change.

The bionic UAAV has typical bionic hydrodynamic characteristics with proven hydrodynamic performance.

### 4.3. Longitudinal Aerodynamic Performance of Air-Balanced Flight

**Figure 15:**
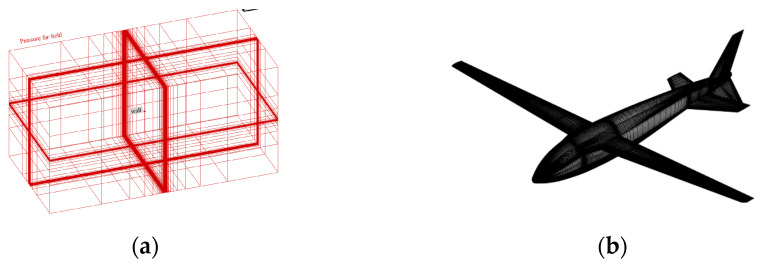
The schematic diagram of bionic UAAV grid division for air-balanced flight. (**a**) Spatial grid; (**b**) airframe grid.

The simulation was carried out for the air flight stage of the bionic UAAV after crossing over the water. The longitudinal aerodynamic calculation was carried out in typical states (altitude below 1000 m, with speeds of 18 m/s and 30 m/s). In this state, the overall configuration is close to a conventional layout aircraft, so the wing chord was used as the characteristic length; the Reynolds numbers were Re1 ≈ 65,000 and Re2 ≈ 110,000, respectively; and the angle of attack for calculation ranged from −4 to 10°. The calculation grid is shown in Figure 15 with the boundary condition of far-field pressure and a fixed non-slip wall.

**Figure 16:**
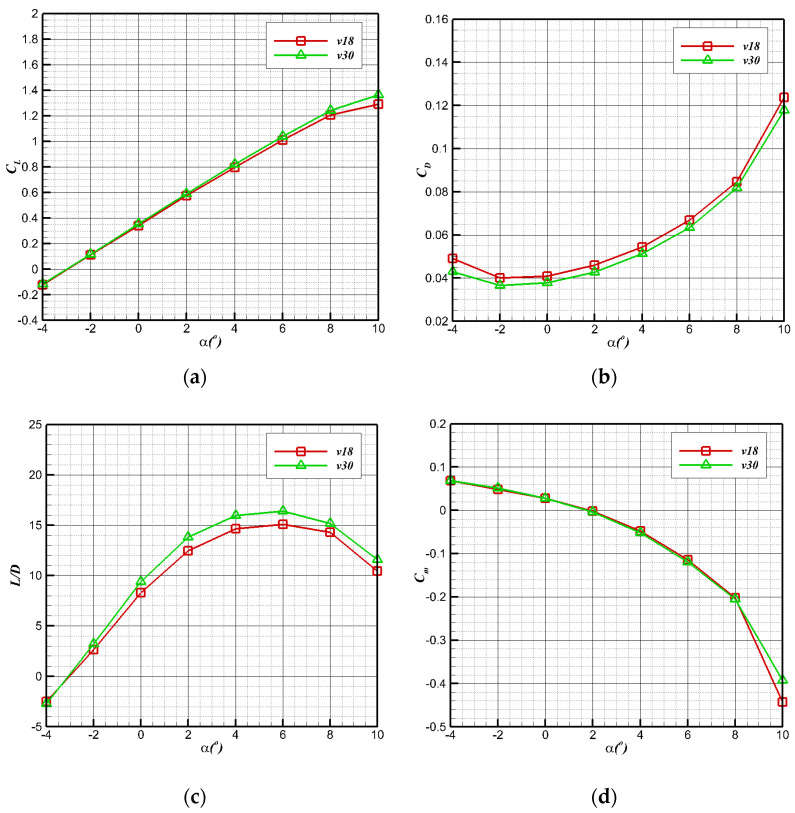
Longitudinal aerodynamic performance of air-balanced flight. (**a**) Lift coefficient; (**b**) resistance coefficient; (**c**) lift–drag ratio; (**d**) pitching moment coefficient.

Figure 16 shows the longitudinal aerodynamic performance under two flight states. In the air cruise stage, the lift coefficient changes linearly with the angle of attack between −4° and 8°. When the angle of attack is greater than 8°, the resistance coefficient increases sharply. The maximum lift–drag ratio is 6°. Under the condition of a low Reynolds number at a speed of 18 m/s, the maximum lift–drag ratio is slightly greater than 15, and the corresponding lift coefficient is about 1, which can provide sufficient lift for air flight. According to the moment characteristics, the pitching moment of the bionic UAAV gradually decreases with the increase in the angle of attack, so we have C_ma_ < 0. Therefore, the bionic UAAV model has longitudinal static stability regarding the air flight stage. The calculation results show that the longitudinal static stability margin ($K_{n}=-\frac{\partial C_{m}}{\partial C_{L}}$) is about 16%.

**Figure 17:**
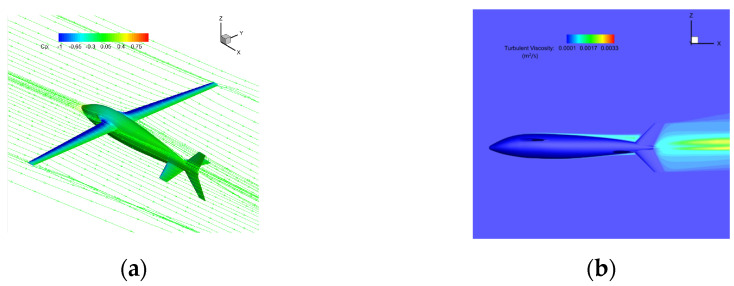
The schematic diagram shows the flow field under a typical air-balanced flight. (**a**) Space streamline; (**b**) surface limit streamline.

Figure 17 shows the flow characteristics of the typical state (with a speed of 30 m/s and an angle of attack of 6°), at which the maximum lift–drag ratio is 16.388. The airflow flows smoothly through the surface of the bionic UAAV model, indicating that the bionic UAAV may have good drag reduction performance. There are whirlpools in the flow at the wing–body fusion part, but there is no separation in the wing spreading direction. The turbulent viscosity strength of the symmetric surface increases significantly along the flow direction, and the tail wings greatly impact the turbulent boundary layer.

### 4.4. Transverse Aerodynamic Performance in Air Flight

**Figure 18:**
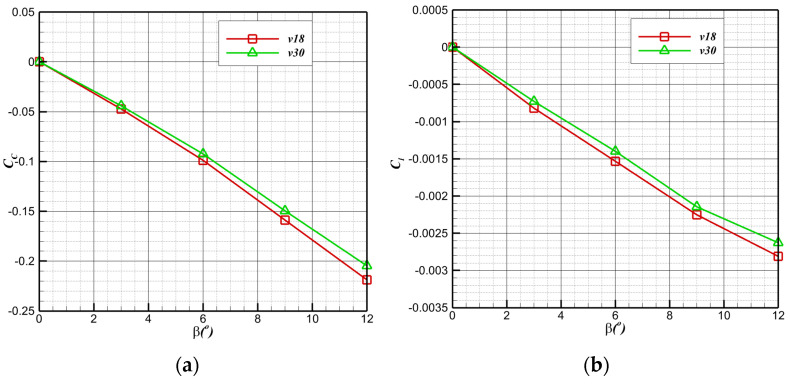

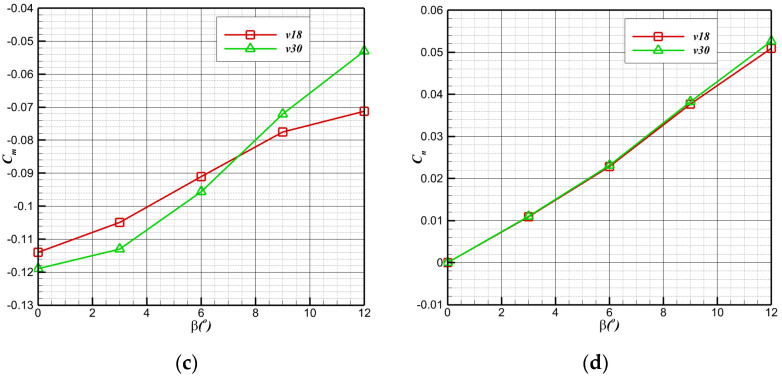
Lateral aerodynamic performance of air-balanced flight. (**a**) Lateral force coefficient; (**b**) roll moment coefficient; (**c**) pitch moment coefficient; (**d**) yaw moment coefficient.

To further evaluate the lateral performance of air flight, the typical state is selected to carry out a lateral aerodynamic calculation (altitude below 1000 m, angle of attack 6°, sideslip angle 0–12°). The lateral aerodynamic performance is shown in Figure 18: the lateral force coefficient ($C_{C}=\frac{C}{\frac{1}{2}\rho {V_{\infty }}^{2}S}$), the yaw moment coefficient ($C_{n}=\frac{C}{qS_{w}b}$), and the roll moment coefficient ($C_{l}=\frac{L_{A}}{qS_{w}b}$) change linearly. The bionic UAAV has essential lateral static stability with the increase in the lateral slip angle (β).

**Figure 19:**
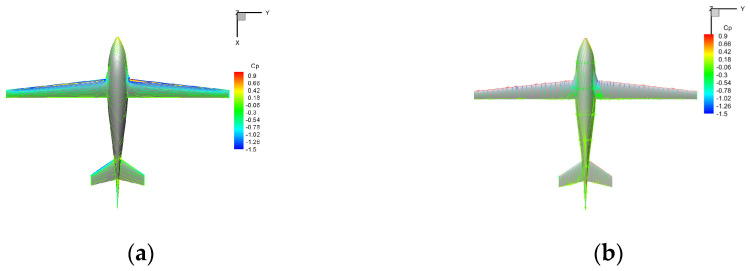
The schematic diagram of flow field under medium sideslip angle in air-balanced flight (v = 30 m/s, α = 6°, β = 6°). (**a**) Pressure distribution; (**b**) surface limit streamline.

The flow field in a typical sideslip flight is shown in Figure 19. The flow strength at the windward side of the wing is more vital than that at the leeward side, resulting in the asymmetry of the pressure distribution on both sides, which has a direct impact on the magnitude of the roll moment. Compared with the case of no sideslip, the vortex generated at the junction of the trailing edge of the wing on the windward side and the fuselage is intensified, the eddy current intensity on the leeward side is reduced, and the airflow separates at the leeward side of the rear part of the fuselage.

**Figure 20:**
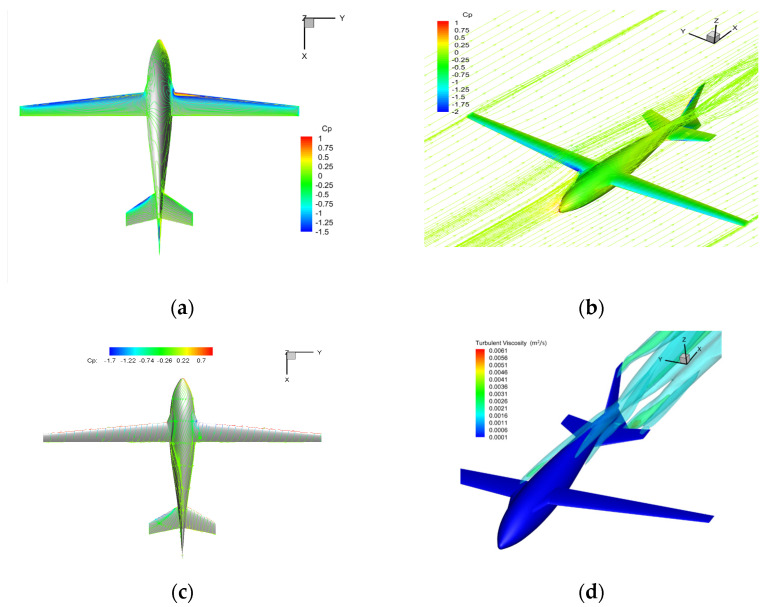
The schematic diagram of flow field under a large sideslip angle in air-balanced flight (v = 30 m/s, α = 6°, β = 12°). (**a**) Pressure distribution; (**b**) space streamline; (**c**) surface limit streamline; (**d**) turbulent viscosity contour.

The typical flow field in a large sideslip is shown in Figure 20. The pressure asymmetry on both sides is intensified, and an apparent tip vortex can be seen at the vertical tail wing. The space and surface limit streamline show that the vortex intensity at the trailing edge of the wing root on the windward side is further increased. In contrast, the leeward side differs from conditions of no sideslip and small sideslip, and no apparent eddy current is seen. In the vertical tail wing leeward region, an apparent wake vortex and significant sideslip flow separation are induced, which may lead to a nonlinear yaw moment.

## 5. Performance of The Water Cross-Over Process and Acceleration Process on the Water

For the process of crossing over the water to attain a stable level flight, the load and pitch aerodynamic and hydrodynamic characteristics are analyzed by numerical simulation, and the aerodynamic characteristics of the acceleration process on the water are evaluated and analyzed.

### 5.1. Load Characteristics of Water Cross-Over Process

The water cross-over angles (θ) of 10°, 20°, 30°, 60°, and 90°, and the water cross-over speeds of 0.5 m/s, 1 m/s, and 2 m/s are selected for the calculation to analyze the hydrodynamic load characteristics of bionic UAAVs.

**Figure 21:**
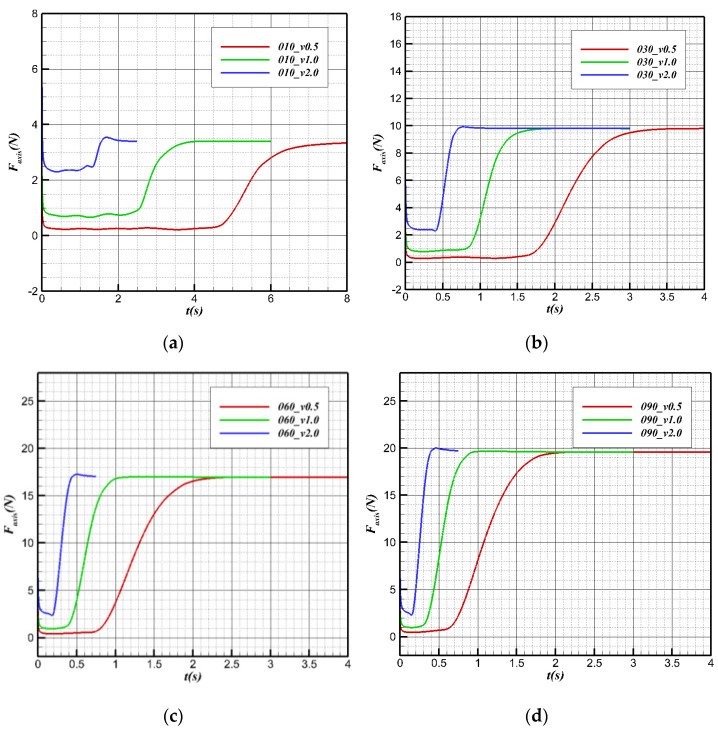
Axial force variation with time during the water cross-over process. (**a**) θ = 10°; (**b**) θ = 30°; (**c**) θ = 60°; (**d**) θ = 90°.

Figure 21 shows the changes in axial forces at different speeds (with a downward direction along the positive body axis). With the assumption that the bionic UAAV can maintain stability before the head exits the water and after the tail leaves the water, the forces along the body axis are almost unchanged before contacting the water and after complete deviation in the uniform rate of the water cross-over process. The larger the water cross-over speed is, the greater the axial force is. When completely out of the water, the differences in axial forces corresponding to different speeds are minimal. This indicates that when the water cross-over speed is less than 2 m/s, the aerodynamic resistance is minimal, and gravity is the main factor determining the magnitude of the axial force. The time required for the water cross-over corresponds to different speeds, and the speed is reciprocal to the time needed for the water cross-over. The smaller the speed is, the more stable the axial force changes in the process. When the speed is 2 m/s, due to the phase transition, the axial force fluctuates when UAAV touches the water surface and ultimately leaves the water surface during the process.

**Figure 22:**
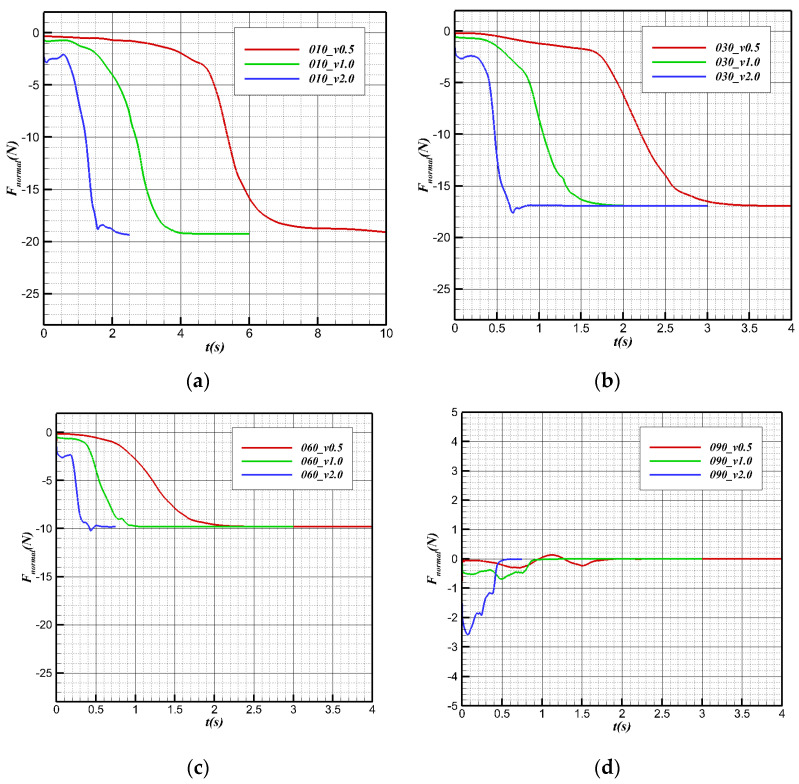
Normal force variation with time during the water cross-over process. (**a**) θ = 10°; (**b**) θ = 30°; (**c**) θ = 60°; (**d**) θ = 90°.

Figure 22 shows the changes in regular forces at different speeds at the same angle (with the positive vertical axis direction). The normal force after complete water cross-over is smaller than the normal force before contact with the surface without the buoyancy provided by water. Before contact with the surface, the buoyancy and gravity remain unchanged, and the normal force of the aircraft is determined only by the force from the water on the plane. Before contact with the surface, the higher the speed is, the smaller the normal force is. After leaving the surface, the buoyancy stops changing, and the normal force almost does not change with speed, mainly affected by the gravity component.

**Figure 23:**
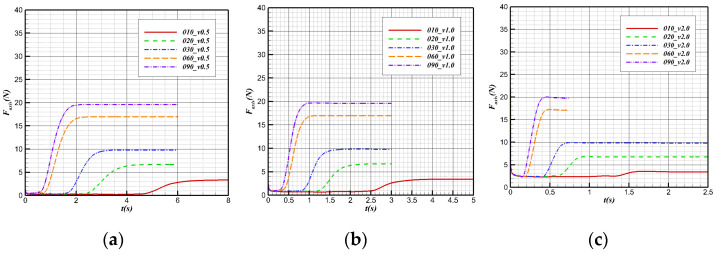
Axial force variation with time at different water cross-over angles. (**a**) v = 0.5 m/s; (**b**) v = 1 m/s; (**c**) v = 2 m/s.

The change in axial force in the water cross-over process at different angles is shown in Figure 23. Before contact with the water surface, the axial forces of the bionic UAAV with different water cross-over angles at the same speed are the same. At this time, the buoyancy is equal to gravity. In the movement process, the equivalent angle of attack of the bionic UAAV is all 0°, and the axial hydrodynamic resistance determines the axial force. The resistance coefficient and the axial force of the same speed are equal.

In the process of the bionic UAAV from initial contact with the water to complete departure from the water, the change in the water cross-over angle will not affect the simulation time of this process, due to the constant body length and constant speed. However, the changes in the axial forces in this process are different due to the other axial forces after the processes corresponding to different water cross-over angles. As the angle increases, the slope of the axial force also increases.

**Figure 24:**
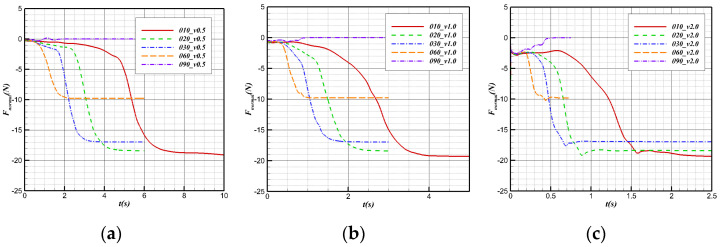
Normal force variation with time at different water cross-over angles. (**a**) v = 0.5 m/s; (**b**) v = 1 m/s; (**c**) v = 2 m/s.

The changes in the regular forces during the water cross-over process at different angles at the same speed are shown in Figure 24. Before contact with the water surface, the normal force of the bionic UAAV is mainly determined by the lift coefficient and speed of the vehicle at the 0° underwater angle of attack. Therefore, the regular forces corresponding to different angles are the same at the same speed. In the water cross-over process, when the water cross-over angle is 10°, 20°, 30°, and 60°, the regular forces all show a downward trend under the same speed because the component of the force of buoyancy and gravity along the vertical axis is the main component of the normal force of the bionic UAAV model. The normal force gradually decreases in this process because of the decrease in the standard buoyancy component. When the water cross-over angle is 90°, because there is no component of the corresponding force of buoyancy and gravity along the vertical axis, the process is mainly determined by the lift provided by the medium. For the water cross-over configuration, the lift coefficient C_L_ corresponding to the 0° angle of attack during underwater navigation is less than 0, so the normal force F_normal_ < 0. Due to the vast difference in physical properties between water and air, the normal force of the vehicle in the air is more significant than that underwater, that is, Fnormal-air > Fnormal-water. Hence, the normal force shows an upward trend.

### 5.2. Pitching Characteristics of the Water Cross-Over Process

Because the pitching characteristics in the water cross-over process have an essential impact on attitude stability, the numerical simulation analysis is conducted to perform the pitching moment in the water cross-over process.

**Figure 25:**
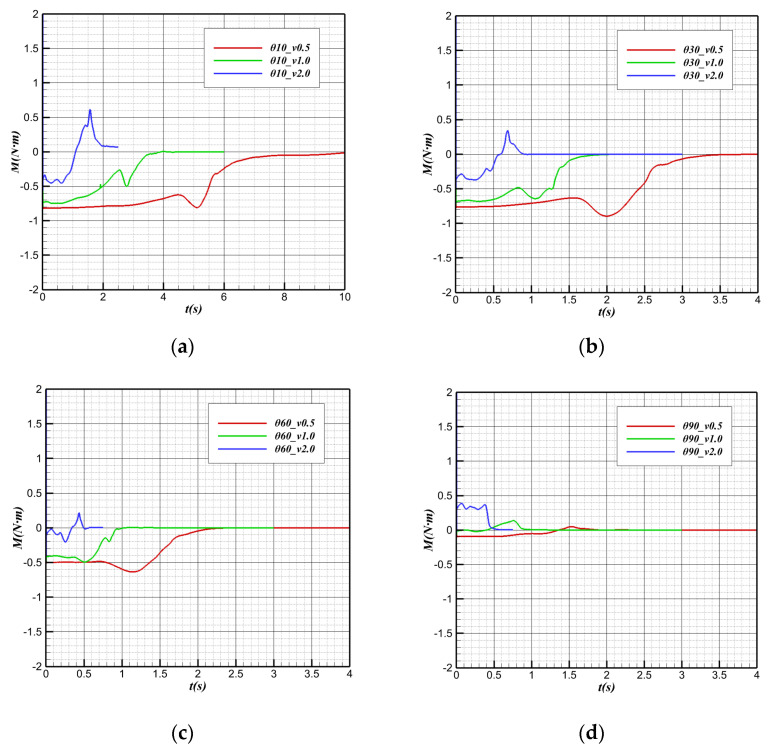
Pitching moment variation with time during the water cross-over process. (**a**) θ = 10°; (**b**) θ = 30°; (**c**) θ = 60°; (**d**) θ = 90°.

Figure 25 shows the changes in the pitching moment in the water cross-over process. The vehicle is subjected to a downward moment in the initial state: the pitching moment M < 0. In the water cross-over process, the vehicle’s low moment gradually decreases and tends to 0 N·m after complete water cross-over.

In the case of the same water cross-over angle, before the bionic UAAV contacts the water, the downward moment decreases with the increase in speed. The center of buoyancy underwater is located after the offset point (center of gravity). In most cases, the downward moment imposed by the buoyancy on the vehicle is numerically more significant than the low moment generated by the relative movement of water. For a constant water cross-over angle, the larger the water cross-over speed is, the larger the upward moment caused by the buoyancy is. When the speed reaches a critical value, the total pitching moment of the vehicle can be 0. Figure 25d show that at a larger water cross-over angle (60°, 90°), when the water cross-over speed is v > 2 m/s, the pitching moment of the vehicle before contacting the water will be greater than 0.

When the water cross-over is with an angle of tilt and the water cross-over angle is the same, the pitching moment has a short sinking stage when the bionic UAAV contacts the water surface, and this phenomenon has signs of weakening with the increase in the water cross-over speed. With the decrease in displacement volume after the aircraft contacts the water surface, the position of the buoyancy center changes. It can be inferred that when the aircraft reaches the water surface, the buoyancy center moves away from the center of gravity in the axial direction, which offsets the decrease in the uplift moment caused by the buoyancy reduction to some extent. At this time, the reduction in the downward moment caused by buoyancy is temporarily suppressed, and the low moment even further increases to some extent. However, with further departure from the water surface, the decrease in the quiet moment caused by the reduction in buoyancy cannot be offset by the gradual increase in pitch moment caused by the relocation of the buoyancy center. Then, the downward moment will gradually decrease until it completely crosses over the water.

After the bionic UAAV leaves the water surface, it is only affected by aerodynamics and gravity, and the point of actuation by gravity is located at the barycenter. Therefore, the pitch moment of the aircraft in the air depends only on the moment imposed by the air during the movement. Since the air density is only 1/1000 of water, the vast difference in the physical properties of the medium leads to the fact that the pitch moment is approximately equal to 0 compared with the pitch moment in the water after completely crossing over the water.

**Figure 26:**
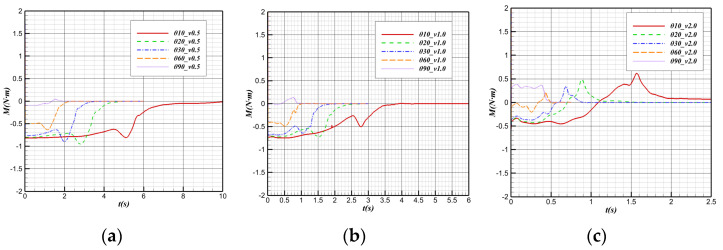
Pitching moment variation with time at different water cross-over angles. (**a**) v = 0.5 m/s; (**b**) v = 1 m/s; (**c**) v = 2 m/s.

At the same water cross-over speed, the variation in the pitch moment with time at different water cross-over angles is shown in Figure 26. It is known that the position of the buoy center is located after the center of gravity in the direction of the body axis, and there is a cosine function between the projection length and angle along the horizontal direction between the buoy center and the center of gravity. That is,

(3) $$X_{h}-X_{g}=\sqrt{{\left(x_{h}-x_{g}\right)}^{2}+{\left(z_{h}-z_{g}\right)}^{2}}\cos\theta$$

With the increase in the water cross-over angle, the horizontal distance between the buoy center point and the center of gravity gradually decreases, which makes the downward moment provided by the buoy gradually decrease.

From the “water mound” phenomenon generated by the bionic UAAV near the water surface to the break of the “water mound”, the pitching moment will sink, and the downward moment will increase. The change in the center of buoyancy in the horizontal direction mainly causes this phenomenon. When the water cross-over angle is large, the horizontal displacement of the buoy center will be much smaller, so when the water cross-over angle is large, the decrease in pitching moment when it touches the water surface will be much weaker. By observing the pitching moment variation curves at the water cross-over speeds of 0.5 m/s and 1 m/s, it can be found that the pitching moment variation at a 60° water cross-over angle is not obvious compared with that at a smaller angle. When the water cross-over is vertical, the moment sinking phenomenon is almost not observed when the “water mound” breaks.

### 5.3. Acceleration Process on the Water

Influenced by the different physical properties of fluids, if the bionic UAAV reaches the takeoff speed immediately after the water cross-over stage, the thrust–weight ratio should be very large, and the load caused by resistance should also be very large. Therefore, the water surface acceleration after the tail leaves the water is an optimal and feasible movement mode of bionic UAAVs.

For the acceleration process on the water, the numerical simulation analysis is carried out to takeoff at an acceleration of 3 m/s^2^. The characteristics of the water–gas phase and load changes in the acceleration process are evaluated and studied.

**Figure 27:**
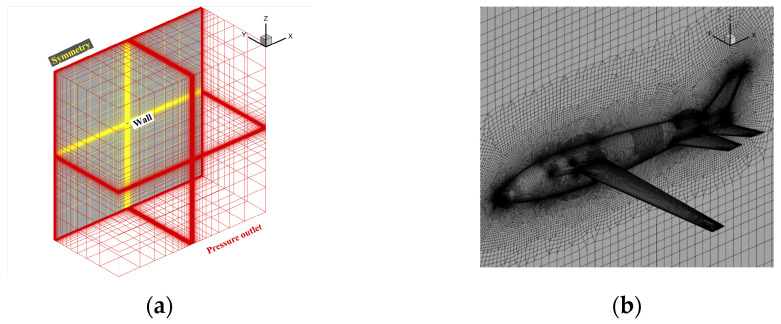
Schematic diagram of water acceleration process on the water. (**a**) Spatial grid; (**b**) symmetrical surface and airframe grid.

The model is divided into a tetrahedron–hexahedron mixed grid in the numerical simulation, the main body of the computing domain is divided into a structural grid, and the unstructured grid is used in the local complex area of the airframe only. At the same time, to reduce the discrete error, the grid near the water–air interface is encrypted to a certain extent. The grid is shown in Figure 27, with far-field pressure and the object surface as a solid wall without a slip boundary condition adopted.

**Figure 28:**
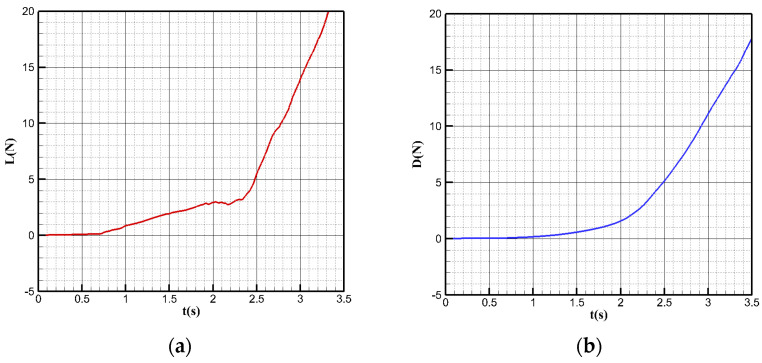
The curve of rising resistance during acceleration. (**a**) Lift change; (**b**) resistance change.

In the acceleration process on the water, the calculated speed is gradually accelerated from zero to tens of meters per second, and lift resistance changes with time for calculation, as shown in Figure 28. Before t = 2.3 s, the lift and resistance grow relatively slowly. After t = 2.3 s, the lift resistance increases significantly. At t = 2.8 s, the lift reaches 10.3 N, and the bionic UAAV achieves the minimum takeoff speed and leaves the water surface. In the acceleration process, the maximum resistance equal to 8.44 N corresponds to the moment before takeoff. That is, the whole thrust of the bionic UAAV should be greater than 8.44 N to meet the needs of acceleration in the process of takeoff from water.

**Figure 29:**
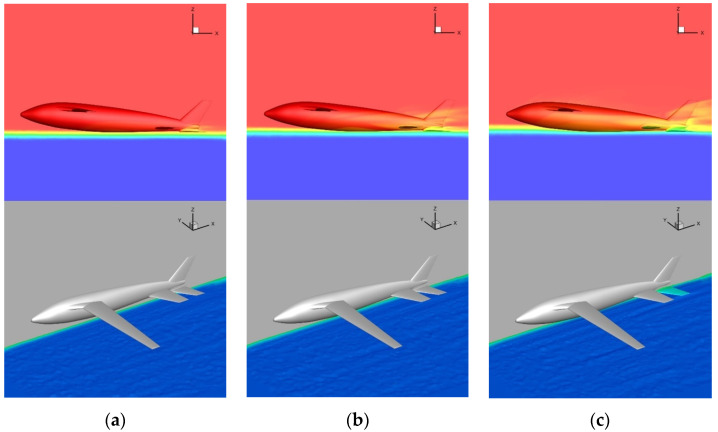
Comparison of phase and pressure distribution at different times. (**a**) t = 1 s; (**b**) t = 2.3 s; (**c**) t = 2.8 s.

Figure 29 shows the corresponding phase changes at different times in the acceleration process on the water. In the early stage of bionic UAAV acceleration, most of the fuselage is air without a water phase, and the water–air boundary is clear. At t = 2.3, the liquid surface is affected by airflow, and part of the water begins to appear on the upper surface of the deformed pendulous tail wings. From this moment on, the load increase trend of the bionic UAAV is significantly intensified. Before t = 2.8 s, the flattened part of the pendulous tail wing is wholly immersed below the water surface, which will produce considerable resistance, and the liquid surface fluctuation near the drooping tail intensifies.

## 6. Water Cross-Over Test Verification

### 6.1. Experimental Device

**Figure 30:**
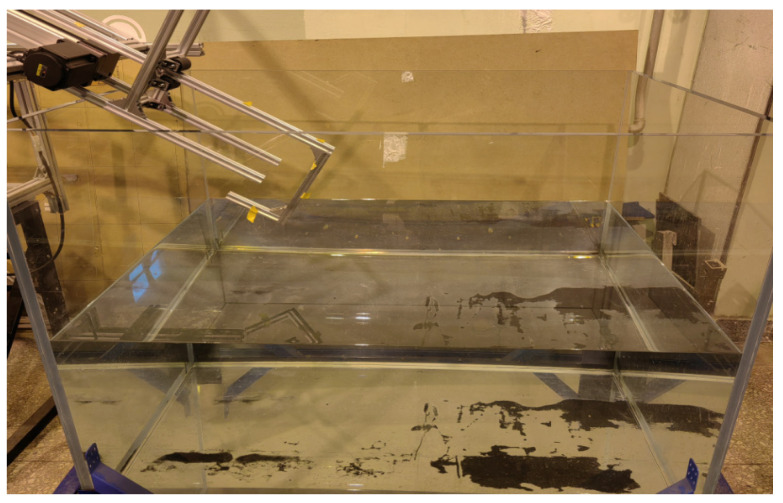
Water storage tank.

An experimental device was set up to verify the principle in the water cross-over process. In the experiment, the water cross-over angle ranges from 10° to 70°, and the water cross-over speed ranges from 0 to 3 m/s. The experiment was completed in the water tunnel laboratory of Beihang University. The water storage tank comprised five pieces of 2 cm thick rigid glass. The bottom has a round drainage hole, and the PVC pipe is connected to the water hole. The water injected into the water storage tank can be discharged by opening the valve. The whole water storage tank is 1.1 m high, 1.5 m long, and 1 m wide, with a maximum water storage capacity of 1.65 tons. The bottom of the water storage tank is fixed on the rectangular frame built by profiles, and four rollers are installed at the bottom of the frame to realize the movement of the water storage tank. The actual picture of the water storage tank is shown in Figure 30.

**Figure 31:**
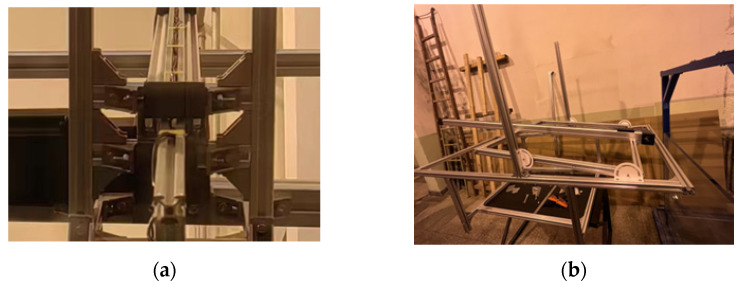
Main components of experimental support. (**a**) Damping structure; (**b**) main body of the bracket.

The water storage tank test device adopts the water cross-over attitude control bracket. The bracket has a primary fixed platform calibrated by the gradient. The bracket can realize the change in the water cross-over angle of 10–70° by adjusting the angle adjustment device, composed of four angle adjustment plates and profiles. The bearing roller pulley is fixed on the bracket near the end of the water cross-over rod with a corner code and double-head bolts, which are used to control the vibration of the bracket in the movement process to reduce the experimental error. The schematic diagram of the test bracket is shown in Figure 31.

**Figure 32:**
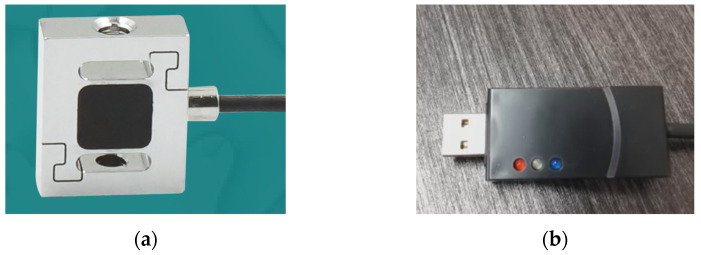
Sensor and data acquisition device. (**a**) AR-DN20 S-type tension and pressure sensor; (**b**) USB06 acquisition card.

The test model was installed on the bracket with a sliding module during the test, and the water cross-over control was carried out. The module was a customized MF60 synchronous sliding module with a precision of 0.05 mm and a built-in linear guide rail of high precision and high load. The design reduction ratio of the sliding module is that the sliding plate moves 150 mm when the motor shaft rotates one time, which needs to be matched with a stepper motor or a servo motor for work. With an 80-servo motor, an LW100 driver, and an HF020 motor controller being used, the movement distance in the controller is consistent with the actual distance. The test sensor used was Allison’s S-type tension and pressure sensor (seen in Figure 32), model AR-DN20. The mass of the scale model used in the experiment was 87.51 g, so the range of the customized sensor was 200 g, with a comprehensive accuracy of 0.1%F.S. With a high-speed data acquisition module, the measured data and curves were packaged into documents and output to the computer for further data processing.

### 6.2. Design of Scale Model and Connection Mechanism

**Figure 33:**
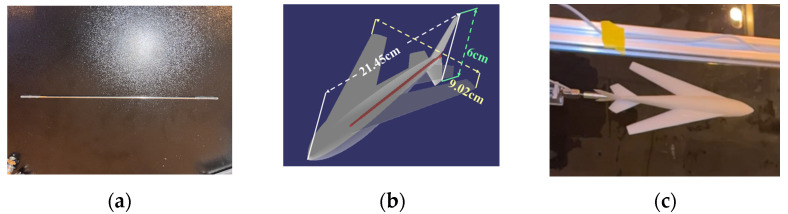
Scale model and connection mechanism. (**a**) Support element; (**b**) scale model; (**c**) connection physical picture.

According to the requirements of bionic UAAV water cross-over verification and the constraints of the test device, the scale model was used to verify the axial force change in the water cross-over process. According to the size of the storage tank, the size of the scaled model is one third of the prototype. The schematic diagram of the scale model and the connection mechanism is shown in Figure 33. To facilitate the force measurement of the sensor, the connector between the scale model and the sensor was separately built in 3D and processed by a CNC. To reduce the vibration of the model perpendicular to the body axis in the water cross-over process after connection, a double-headed bolt with a diameter of 2 mm and a length of 150 mm was used as a support to penetrate the rear part of the model and connected the model with the connector. This connection scheme modified the scale model, including the double-headed bolt, CNC-processed connector, modified scale model, and connection. Finally, the scale model was 3D-printed by 9000R SLA photosensitive resin, with a size of 21.45 × 9.02 × 6 cm and a mass of 87.51 g.

### 6.3. Experimental Results and Analysis

#### 6.3.1. Water Cross-Over Phase Change of the Scale Model

**Figure 34:**
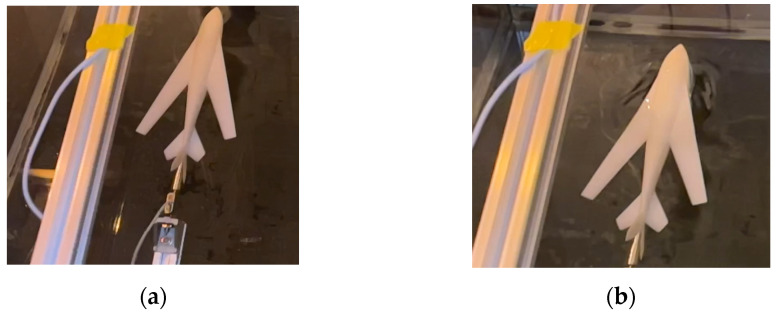

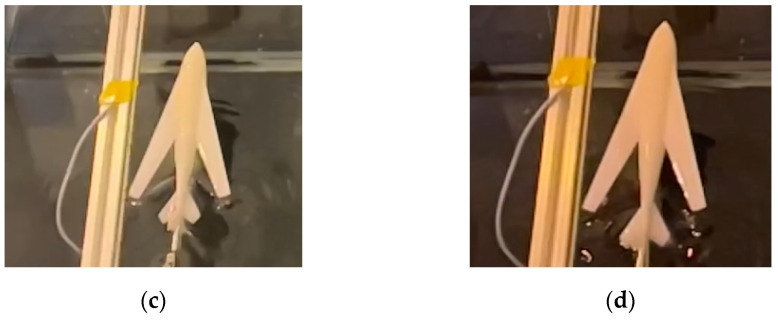
Waterbody response in the typical stage of slight water cross-over angle. (**a**) Close to the water surface; (**b**) the nose leaves the water; (**c**) the wing goes into the water; (**d**) the tail goes into the water.

Since the water cross-over process is accompanied by the water–gas phase change, to observe the phase change better, a slight water cross-over angle of 20° and a small water cross-over speed $\sqrt{3}$/6 m/s were selected to analyze the water response and phase change in the water cross-over experiment. The duration of the model from contacting the water surface to leaving the water surface altogether was about 0.7 s. Taking the distance between the model head and the water surface as a reference, four typical states were selected for analysis. Figure 34 shows the corresponding images of water at different stages of slight water cross-over angles. When the model is close to the water surface, the water surface near the nose produces a “water mound”, which is consistent with the simulation phenomenon. As the model continues to move forward, the liquid level near the fuselage rises, the liquid is brought out of the water by the model, and the water surface near the model fluctuates obviously (referring to Figure 34b). When the wing leaves the water surface, the apparent “water curtain” can be observed at the rear end of the wing (referring to Figure 34c). When the wing ultimately leaves the water, the tail begins to leave the water. The water curtain breaks, and the water from the wing gathers along the trailing edge. Then, it drops into the pool, and the water surface fluctuates violently at the point where the liquid drops into the water (referring to Figure 34d).

#### 6.3.2. Changes in Axial Forces of the Scale Model

**Figure 35:**
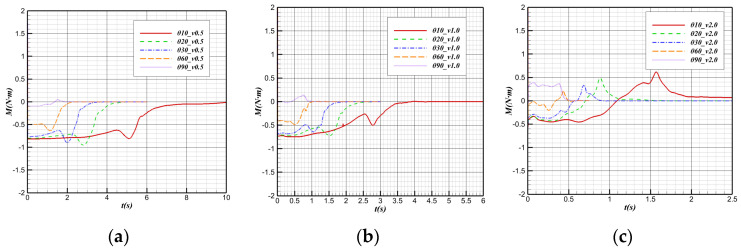
Comparison curve of axial force variation between experiment and simulation. (**a**) v = 0.5 m/s; (**b**) v = 1 m/s; (**c**) v = 2 m/s.

The mass of the scale model itself is 87.51 g, and the total weight of CNC processing connectors, screws, and nuts is 91.388 g, with 73.63 g corresponding to the weight of the scale model. Therefore, after collecting the data through the high-speed data acquisition device, the data need to be processed first. The sensor unit used is g, and after converting it into N and subtracting the extra-axial component of the gravity of the measurement model, the data obtained are the final data to be compared with the simulation. The size of the model used in the experiment is 1/3 of the original model, based on flow similarity theory and criteria; its movement time is 1/$\sqrt{3}$ times the simulation time; and its axial force should be close to 1/27 times the simulation in value. The axial force changes with time obtained by adjusting the range of the coordinate axis correspondingly and finally, as shown in Figure 35.

The experimental data and simulation results are consistent in trend, and the corresponding proportion of the value is also in line with the previous analysis. The axial force obtained by the sensor before contacting the water surface changes smoothly. At the experimental angle of 27° and v = 1.155 m/s, which is the water cross-over speed, there is a significant oscillation in the axial force change after the model leaves the water. Since the model and the sensor are not ideal rigid bodies after being connected, the fixed point is designed at the end of the model to reduce the errors caused by the water disturbance in the experiment. Therefore, after the model is out of the water, due to the significant change in the physical properties of the medium, the model will have a short jitter in the average direction, and the smaller the angle, the more intense it will be. Although the overall axial force fluctuates near the theoretical value after treatment, it can still be used to verify the above simulation results.

The experimental and simulation results generally agree with the axial force, especially after the model is entirely out of the water, and the axial force before contacting the water is slightly different from the simulation results.

## 7. Conclusions

(1) The bionic conceptual design of crossing over water is excellent by planning multiple stages and modes of motion. It can fully integrate flying fish features and bionic UAAV flight requirements of water cross-over.
(2) The bionic UAAV, modeled as a flying fish, has an excellent variant configuration to adapt to water cross-over. It has a high essential aerodynamic and hydrodynamic performance. Its navigation stability is good, including longitudinal and lateral stability during water and air navigation. The cruise-flying lift–drag ratio is greater than 15 at a low Reynolds number.
(3) The axial impact load of the bionic UAAV regularly increases with the angle and velocity. The pitching moment has a “water mound” forming and breaking when the bionic UAAV moves from the water–air interface to away from the water surface. These characteristics can work together with acceleration to fly near the water’s surface to achieve a normal process of outflowing water.
(4) The phase and axial force of the water cross-over experiment and simulation can be agreed upon. The present method and the bionic variant configuration provide a feasible water cross-over design and analysis strategy for bionic UAAVs.
(5) The present UAAV has bionic water cross-over and inverse kinematics characteristics of robotics. It is expected to have wide application in military and civilian fields, with the joint development of modern mechanical modeling and simulation methods related to Industry 4.0, multi-layer sensing systems, and airborne navigation systems.

## Figures and Tables

**Table 1:** Fuselage contour parameter.

Parameter Type Value	Parameter Type Value
Length/m	0.606
Width/m	0.041
Height/m	0.091

**Table 2:** Configuration parameters of underwater diving and water cross-over processes.

Part	Parameter Type	Value
Wing	Span/m	0.239
	Exposed area/m^2^	0.0326
	Chord length/m	0.212
Horizontal tail	Span/m	0.09439
	Exposed area/m^2^	0.0067
Vertical fin	Span/m	0.18
	Exposed area/m^2^	0.009

**Table 3:** Configuration parameters of acceleration process on the water.

Part	Parameter Type	Value
Wing	Span/m	0.834
	Exposed area/m^2^	0.037
	Chord length/m	0.07
Horizontal tail	Span/m	0.2
	Exposed area/m^2^	0.011
Upper vertical fin	Span/m	0.09
	Exposed area/m^2^	0.0045
Lower vertical fin	Span/m	0.145
	Exposed area/m^2^	0.0072

**Table 4:** Configuration parameters of acceleration process on the water and air-balanced flight.

Part	Parameter Type	Value
Wing	Span/m	0.834
	Exposed area/m^2^	0.037
	Chord length/m	0.07
Horizontal tail	Span/m	0.2
	Exposed area/m^2^	0.011
Vertical fin	Span/m	0.18
	Exposed area/m^2^	0.009

## Data Availability

The authors do not have permission to share data.
